# Layering Methylome and Transcriptome in the Same Tissue Slice

**DOI:** 10.1093/gpbjnl/qzaf136

**Published:** 2026-01-12

**Authors:** Yang Xiao, Sai Ma

**Affiliations:** Department of Pathology, University of Michigan Medical School, Ann Arbor, MI 48109, USA; Department of Computational Medicine and Bioinformatics, University of Michigan Medical School, Ann Arbor, MI 48109, USA; Department of Biomedical Engineering, University of Michigan, Ann Arbor, MI 48109, USA; Department of Genetics and Genomic Sciences, Icahn School of Medicine at Mount Sinai, New York, NY 10029, USA; Icahn Genomics Institute, Icahn School of Medicine at Mount Sinai, New York, NY 10029, USA

Understanding how DNA methylation regulates gene expression within the native spatial context remains a long-standing technical challenge. Single-cell methylome profiling has provided important insights into cell identity and epigenetic mechanisms, but the employed approaches require physical dissociation of tissues, and thus sacrifice complex spatial organization [1,2]. Moreover, because methylome and transcriptome measurements are typically obtained from separate assays or distinct cells, the direct link between epigenetic state and gene expression outcome is lost.

A new study led by the groups of Drs. Yanxiang Deng and Wanding Zhou at the University of Pennsylvania, entitled “*Spatial joint profiling of DNA methylome and transcriptome in tissues*” [3], developed a breakthrough technology that address these gaps. This novel method, termed Spatial-DMT, enables simultaneous *in situ* detection of DNA methylation and mRNAs from the very same tissue slice, preserving both spatial and molecular information at near single-cell resolution (up to 10 μm pixel resolution). By co-registering methylome and transcriptome, Spatial-DMT provides a powerful platform for mapping how local DNA methylation states correspond to gene expression programs across developmental stages.

Spatial-DMT lies in its use of microfluidic channels as miniature bioreactors, enabling precise execution of sequential biochemical reactions *in situ* [4,5]. The microfluidic setup allows spatial indexing and compartmentalization of nucleic acids while preserving the native tissue architecture (**Figure 1**A). Following permeabilization and molecular barcoding, genomic DNA (gDNA) and cDNA are physically separated, allowing independent yet spatially linked processing of the methylome and transcriptome. The transcriptome is captured through reverse transcription and template switching, while gDNA undergoes enzymatic methylation sequencing (EM-seq) [6]. In the EM-seq process, methylated cytosine (5mC) is oxidized by an enzyme, ten-eleven translocation 2 (TET2), to 5-carboxylcytosine (5caC), making it resistant to deamination. In contrast, unmethylated cytosine is deaminated by an enzyme, apolipoprotein B mRNA editing enzyme catalytic subunit 3A (APOBEC3A), to uracil, which is subsequently read as thymine during sequencing. As a result, 5mC remains identifiable as cytosine, while unmethylated cytosine is detected as thymine. This allows for accurate distinction between methylated and unmethylated cytosines, facilitating high-resolution readout of methylation states in tissues. Downstream splint ligation and template switching introduce sequencing adapters and PCR handles, ensuring efficient library amplification. Together, this workflow enables multi-step enzymatic reactions to be carried out *in situ*, generating high-quality spatial maps that integrate both transcriptional and epigenetic information (Figure 1B and C).

**Figure 1:**
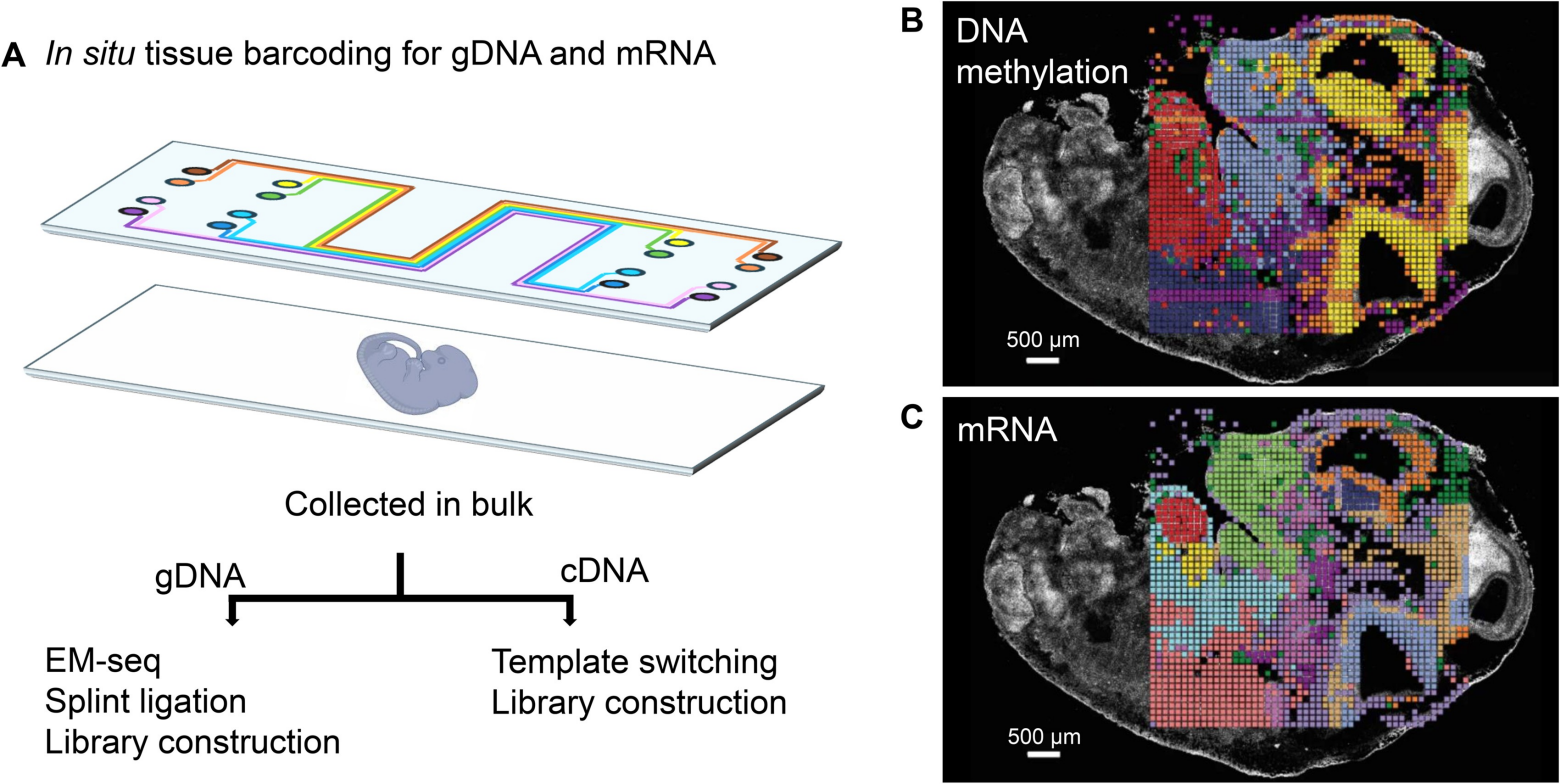
Schematic of spatial joint profiling of the DNA methylome and transcriptome **A**. Using microfluidic channels, spatial indexes are added to DNA or mRNA molecules during the *in situ* barcoding process. Mouse embryos at embryonic day 11 were simultaneously profiled for DNA methylation (**B**) and mRNA (**C**). Pixel size of detection is 50 µm. Scale bar: 500 µm. The schematic is adapted from [3] with CC-BY 4.0. gDNA, genomic DNA; EM-seq, enzymatic methylation sequencing.

Spatial-DMT is made possible by several elegant molecular innovations. First, it employs EM-seq, a strategy that avoids the harsh degradation associated with bisulfite treatment and is thus well suited for low-input, spatially barcoded material. Second, it adapts the VeraSeq indexed PCR strategy, ensuring that DNA fragments remain amplifiable even after cytosines in the PCR handles are converted to thymines. Third, it leverages HCl treatment to effectively remove histone proteins, enabling more uniform genomic coverage. Together, these innovations allow Spatial-DMT to achieve near single-cell spatial resolution (10–50 μm), which is a remarkable technical advance. As with any pioneering technology, there remain opportunities for refinement. The number of CpG sites detected is currently lower than that in single-cell DNA methylation assays, conversion efficiency is not yet complete, and the number of genes captured in the RNA-seq assay is modest compared to other single modality spatial transcriptomics methods. These challenges underscore exciting directions for further methodological optimization, from enhancing conversion chemistry to improving capture efficiency and transcript recovery. Despite these current limitations, Spatial-DMT represents an important milestone: it is the first demonstration that spatial DNA methylation can be jointly profiled alongside gene expression. When paired with spatial transcriptome profiling, it not only deepens our understanding of gene regulation but also opens unprecedented opportunities to interrogate how molecular programs are organized in space and time. This work has the potential to transform studies of aging, cancer, and neurological disorders, where DNA methylation plays central and dynamic roles. With continued innovation, improvements in data quality and throughput are likely to follow rapidly, paving the way for increasingly comprehensive and integrated spatial epigenomic maps.

Applying spatial-DMT to developing mouse embryos at two key developmental stages (embryonic days 11 and 13), and to postnatal mouse brain, the authors generate detailed “bimodal” tissue maps that capture both epigenetic and transcriptomic landscapes. Spatial methylome maps reveal features that cannot be captured by dissociated single-cell methods, including mitotic history and region-specific epigenetic variation. These maps reveal how methylation (including CpG and non-CpG methylation) and gene expression vary not just with cell type, but with spatial position and developmental time. Terminally differentiated neural cells exhibit broader methylated domains, consistent with their stable transcriptional programs, whereas progenitor-rich regions such as the subventricular zone display less extensive methylation, reflecting a more stem-like, plastic state. Spatial methylome demonstrates the role of epigenetic modification in stabilizing regional neuronal identity. The integrated analyses uncover previously under-appreciated relationships between methylation states and transcription, including both the expected negative correlations, and also positive ones in certain contexts. Spatial-DMT delineates regional epigenetic dynamics and tissue-specific regulatory programs, all *in situ*.

Looking ahead, addressing challenges such as data sparsity, batch effects, and the complexity of diverse methylation contexts [methylated cytosine in a CG dinucleotide context (mCG), methylated cytosine in a non-CG context (mCH), and hydroxymethylation] will catalyze innovation in computational frameworks. Adapting and extending single-cell analysis strategies to the spatial domain will be key for spatial data analysis, but ultimately new approaches designed specifically for the spatial epigenome may emerge. Beyond methodological advances, spatial DNA methylation adds a transformative dimension to the growing universe of spatial omics modalities, including RNA, chromatin accessibility, histone modification, and protein. Integrating these layers will make it possible to build multi-omics maps of gene regulation *in situ*, revealing how epigenetic states interact with transcriptional programs to shape tissue architecture and cellular function.

In the long term, such integrative spatial maps could move beyond discovery to impact translational research and clinical practice. They may enable the identification of spatial epigenetic biomarkers for early disease detection, provide insights into tumor heterogeneity that inform precision oncology, and uncover spatial epigenetic mechanisms underlying brain function and dysfunction. By bridging molecular regulation with tissue context, Spatial-DMT lays the groundwork for a new era of spatial epigenomics with the potential to reshape both basic biology and medicine.

## CRediT author statement

**Yang Xiao:** Conceptualization, Writing – original draft, Writing – review & editing. **Sai Ma:** Writing – original draft, Writing – review & editing. Both authors have read and approved the final manuscript.
